# Supplementary material for: Chemical Composition of Thymus leucotrichus var. creticus Essential Oil and Its Protective Effects on Both Damage and Oxidative Stress in Leptodictyum riparium Hedw. Induced by Cadmium
Source: Plants (Basel). 2022 Dec 15;11(24):3529. doi: 10.3390/plants11243529 (PMC9785703; doi:10.3390/plants11243529)
Supplement: Supplementary file 1 [file plants-11-03529-s001.zip › plants-2054882-supplementary.pdf]

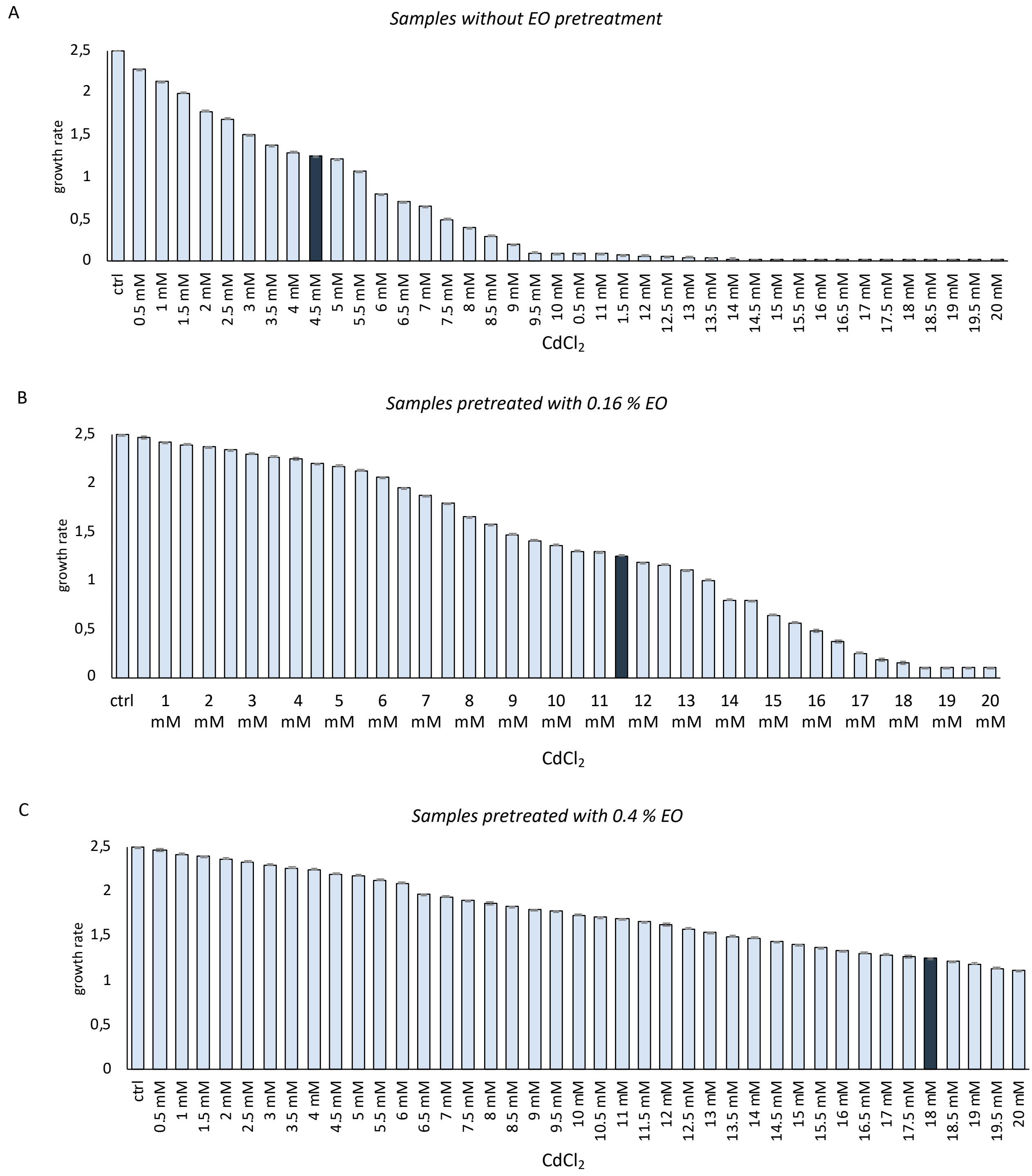

Figure S1. Cadmium toxicity tests. Growth rate values to cadmium concentrations between 0.5 and 20 mM with a progressive increase of 0.5M in *L. riparium* gametophytes treated without EO (A), 0.16% (B) and 0.4% (C) of EO.
